# Meloxicam in Combination with Mitoxantrone or Vinblastine as First-Line Treatment for Non-Resectable Urothelial Cell Carcinoma in Dogs

**DOI:** 10.3390/vetsci10080529

**Published:** 2023-08-21

**Authors:** Estel.la Ciriano Cerdà, Alenka Lavra Zajc, Riccardo Finotello, Kirsty Macdonald, Filipa Lyseight, Nele Van Den Steen, Katia Sanchez Gonzalez, Mary Marrington, Jessica Grant

**Affiliations:** 1 Northwest Veterinary Specialists, Part of Linnaeus Veterinary Limited, Ashville Point, Beechwood, Sutton Weaver, Runcorn WA7 3FW, UK; 2 Department of Small Animal Clinical Sciences, Department of Veterinary Anatomy Physiology and Pathology, Institute of Infection Veterinary and Ecological Sciences, University of Liverpool, Chester High Rd, Neston CH64 7TE, UK; riccardofi@libero.it (R.F.);; 3 Dick White Referrals, Station Farm, London Road, Six Mile Bottom, Cambridgeshire CB8 0UH, UK; 4 Cave Veterinary Specialists, George’s Farm, West Buckland, Nr. Wellington TA21 9LE, UK; 5 Southern Counties Veterinary Specialists, Forest Corner Farm, Hangersley, Ringwood BH24 3JW, UK

**Keywords:** bladder cancer, canine, chemotherapy, COX inhibitor, oncology

## Abstract

**Simple Summary:**

Meloxicam is a readily available non-steroidal anti-inflammatory medication that is commonly used in clinical practice. It has been associated with a low incidence of gastrointestinal adverse effects in comparison to other non-steroidal anti-inflammatory medications and it has also demonstrated anti-tumour activity in vitro. In this study we describe the efficacy of meloxicam in combination with either mitoxantrone or vinblastine as a first-line treatment for non-resectable canine urothelial cell carcinoma (UCC), as well as the incidence of gastrointestinal adverse effects associated with this treatment combination. The results of this study suggest that meloxicam in combination with mitoxantrone or vinblastine, as a first-line treatment for non-resectable UCC, is well-tolerated and potentially effective in inducing cancer response.

**Abstract:**

Cyclooxygenase (COX) inhibitors have been demonstrated to have antitumour activity in canine urothelial cell carcinoma (UCC), given as a sole treatment or in combination with chemotherapy. The purpose of this retrospective multi-institutional study was to assess the efficacy of meloxicam in combination with mitoxantrone or vinblastine as a first-line treatment for non-resectable canine UCC. Gastrointestinal adverse effects (AEs) of these treatment combinations were also assessed. A total of 28 dogs met the inclusion criteria, 21/28 dogs received mitoxantrone and meloxicam, and 7/28 received vinblastine and meloxicam. Tumour response (TR) and AE were evaluated according to Veterinary Co-Operative Oncology Group (VCOG) criteria. The endpoint of the study was the time to tumour progression (TTP). The mitoxantrone-group induced 24% partial response and 62% stable disease, while the vinblastine-group induced 14% and 86%, respectively. Median TTP was 84 days (mitoxantrone and meloxicam, 70 days; and vinblastine and meloxicam, 178 days). The presence of metastatic disease significantly decreased TTP (*p* = 0.007). Gastrointestinal AEs were reported in 21.4% of the patients, with the most common being VCOG grade 1–2 diarrhoea. Meloxicam is a well-tolerated NSAID when combined with mitoxantrone or vinblastine as first-line treatment for non-resectable canine UCC.

## 1. Introduction

Urothelial cell carcinoma (UCC) is the most common neoplasm of the urinary tract in dogs [1,2]. Multiple treatment modalities have been investigated over the years for the management of canine UCC, such as surgery, systemic chemotherapy, radiation therapy, and cyclooxygenase (COX) inhibitors [1].

Non-steroidal anti-inflammatory drugs (NSAIDs) are primarily used for their inflammatory and analgesic effects by blocking the production of prostaglandins (PGs) from arachidonic acid (AA) via inhibition of cyclooxygenase-1 and -2 (COX-1 and -2) activities [3,4,5,6]. They have also demonstrated antitumour activity against a variety of tumour types, including urothelial cell carcinoma [7,8].

It is unclear if the antineoplastic effect of NSAIDs is due to the inhibition of both COX-1 and COX-2 enzymes, or through preferential inhibition of one over the other, especially COX-2. The COX-1 enzyme is constitutively expressed in most tissues, and it is primarily responsible for the protection of the gastric mucosa, maintenance of the renal blood flow, and regulation of platelet aggregation. In contrast, COX-2 expression is normally present in low levels, although it is rapidly induced by inflammatory conditions and neoplastic transformation. Increased COX-2 expression stimulates angiogenesis, inhibits apoptosis, and induces tumour progression and metastasis [7].

The role of the COX-2 enzyme in tumourigenesis and its overexpression in canine UCC [8] has encouraged further investigation into the use of different NSAIDs (COX inhibitors) for the treatment of canine UCC [9,10,11,12].

In the first study assessing naturally occurring tumours treated with piroxicam, a non-selective COX-2 inhibitor, as a sole therapy, reported a partial response (PR) in 13% of the patients. Three of the ten dogs exhibiting partial response were diagnosed with UCC [9]. A second clinical trial assessing piroxicam for treatment of canine bladder UCC described an objective response (OR) (complete response and partial response) of 18% [10]. Deracoxib and firocoxib, selective COX-2 inhibitors, were also evaluated in canine UCC. These showed similar tumour responses to piroxicam with a PR of 17% and 20%, respectively [11,12], but with a shorter tumour time to progression (TTP). The incidence of gastrointestinal adverse effects (AEs) was reported to be 17.6% with piroxicam, 19% with deracoxib, and 33% with firocoxib [10,11,12].

Multiple studies have also assessed the response of different NSAIDs in combination with systemic chemotherapy for the treatment of UCC. The combination of cisplatin with piroxicam or firocoxib had promising tumour responses with clinical benefit (CB) (complete response, partial response, and stable disease) observed in 67–100% of patients. However, these treatment combinations were associated with significant AEs. Marked renal toxicosis (>75%) was the main AE reported with the cisplatin and piroxicam combination [13,14]. Lower doses of cisplatin were assessed to minimise this AE but minimal treatment efficacy and severe renal toxicosis (29%) were noted [15]. Combining this chemotherapy agent with firocoxib was associated with marked renal (45%) and gastrointestinal AEs (66%) [12].

Other commonly used chemotherapy agents for the treatment of canine UCC, vinblastine, carboplatin, and mitoxantrone, were also evaluated in combination with piroxicam. Vinblastine alongside piroxicam showed a CB of 92%, with gastrointestinal AEs reported in 21% of the patients. [16]. Carboplatin and mitoxantrone in combination with piroxicam had a CB of 67–83% [17,18] and 77–81% [18,19], respectively. Most commonly reported AEs were gastrointestinal, which were higher in those patients treated with carboplatin and piroxicam (74%) than those treated with a mitoxantrone and piroxicam combination (18%) [17,18,19]. The latter demonstrated a similar incidence of gastrointestinal AEs to those reported with piroxicam alone [10].

Gemcitabine, doxorubicin, and chlorambucil have also been assessed in combination with piroxicam, showing a CB of 29%, 70%, and 70%, respectively [20,21,22]. Despite tumour responses being lower than those previously reported with platinum-based protocols, mitoxantrone and vinblastine, these treatment modalities could be considered as an alternative treatment strategy for canine UCC associated with minimal side effects. 

In general, the use of chemotherapy in combination with NSAIDs for the treatment of canine UCC showed better tumour responses than NSAIDs alone, but these combinations have been associated with more AEs [9,10,11,12,13,14,15,16,17,18,19,20,21,22].

Meloxicam, a selective COX-2 inhibitor, has shown anti-proliferative and pro-apoptotic effects on canine cancer cells in vitro [23] and in vivo [24,25]. A recent retrospective study was published evaluating meloxicam as a sole therapy or in combination with chemotherapy for the treatment of canine UCC [26]. The median survival time (MST) of the patients treated only with meloxicam (151 days) was similar to that reported with piroxicam alone (181 days) [9,26]. The patients treated with meloxicam in combination with mitoxantrone, occasionally followed by metronomic chlorambucil, had slightly shorter MST (217 days) than previously reported with piroxicam in combination with mitoxantrone (291 days) [18,26]. The time to progression, CB, or the incidence of AEs from this combination treatment were not assessed. Nevertheless, these findings indicate meloxicam could be a good alternative COX-2 inhibitor for the management of canine UCC. The main goal of this retrospective study was to assess the efficacy of meloxicam (COX-2 inhibitor) in combination with either mitoxantrone or vinblastine as a first-line treatment for non-resectable canine UCC, as these are the most commonly used protocols in our centres due to their demonstrated clinical benefit, which is associated with a good toxicity profile. The severity of gastrointestinal AEs associated with this treatment combination was also assessed.

## 2. Materials and Methods

Medical records of dogs diagnosed with nonresectable UCC were retrospectively reviewed from the database of five veterinary referral hospitals (Northwest Veterinary Specialists; Small Animal Teaching Hospital, Liverpool University; Dick White Referrals; Cave Veterinary Specialists; and Southern Counties Veterinary Specialists) in the UK, between January 2010 and February 2022. 

Inclusion criteria limited enrolment to dogs (1) that had cytologically and/or histopathologically confirmed diagnosis of UCC of the bladder, urethra, or prostate; (2) that received either mitoxantrone or vinblastine as a first-line treatment in combination with meloxicam; (3) that had at least initial staging with abdominal ultrasound; (4) for which meloxicam was the only NSAID administered; and (5) for which meloxicam administration was started no longer than 6 weeks prior to commencing chemotherapy treatment. Patients pre-treated with other COX inhibitors were excluded from the study, as well as those patients diagnosed with prostatic carcinoma. 

Dogs were staged according to World Health Organization criteria for urinary bladder tumours [27]. Tumour response was assessed based on ultrasonographic changes using the Veterinary Cooperative Oncology Group (VCOG) consensus document for evaluation response criteria for solid tumours in dogs (RECIST) [28] or based on amelioration or deterioration in clinical signs. The intended restaging schedule consisted of a focal abdominal ultrasound of the urinary tract every 12 weeks, as a minimum, or earlier in the case of deterioration in clinical signs. Treatment-related AEs were classified according to the VCOG common terminology criteria for AEs [29]. 

The endpoint of the study was the time to progression of the patients receiving mitoxantrone or vinblastine in combination with meloxicam. TTP was defined as the time between the first chemotherapy treatment and ultrasonographically documented progression of the disease. Patients who had stable disease or died of causes unrelated to progression of the disease were censored.

For statistical analysis, dogs were divided into 2 groups: the “mitoxantrone-group” (patients treated with a combination of mitoxantrone and meloxicam) and the “vinblastine-group” (patients treated with a combination of vinblastine and meloxicam). Continuous data are reported as medians and ranges, and categorical data as frequencies and percentages. These are summarised for the entire sample, and separately for the two treatment groups. Categorical data (treatment responses and AE) were compared between these groups using the Fisher exact test, and continuous data (characteristics of the sample population) using the Mann Whitney test. The time to progression (TTP) was compared between treatment groups, as well as between metastatic and non-metastatic patients, using Kaplan–Meier survival curves and log rank tests. Analysis was undertaken in Minitab 19 (Minitab LLC, State College, PA, USA) and R 3.6.2 (R Core Team. Vienna, Austria). Statistical significance was considered if *p* < 0.05.

## 3. Results

Twenty-eight dogs met the inclusion criteria. The mean age at diagnosis was 10.5 years (range 4.8–15.1 years). There were six (21.4%) mixed-breed dogs; four (14.2%) Labrador Retrievers; three (10.7%) Scottish Terriers; two (7.1%) English Cocker Spaniels; two (7.1%) West Highland White Terriers; two (7.1%) Shetland Sheepdogs; and one (3.6%) each of Belgian Shepherd dog, Bichon Frise, Cairn Terrier, Cavalier King Charles, English Spring Spaniel, German Shepherd dog, Jack Russel, Miniature Poodle, and a Northern Inuit. There were 11 (39%) spayed females, 16 (57%) castrated males, and 1 (4%) intact male. The mean body weight at the initial presentation was 12.5 kg (range 3.7–39.5). There were no statistically significant differences among groups concerning breed, age, sex, or weight. The characteristics of the two groups are summarised in Table 1.

Eleven dogs had a histopathologically confirmed diagnosis of UCC, and the remaining dogs were diagnosed cytologically via bladder/urethral wash or traumatic catheterisation. The most common clinical signs were pollakiuria (82%), stranguria (75%), and urinary incontinence (14%). Haematuria (32%) and faecal tenesmus (14%) were also reported. One patient was presented with partial urinary obstruction, and in another patient the diagnosis was incidental, discovered during a workup for lethargy.

Full staging including imaging of the thorax and abdomen was performed in 82% (23/28) of the patients and the remaining patients underwent abdominal ultrasound only (three of the mitoxantrone-group and two of the vinblastine-group). Routine urine analysis and culture were not performed in all the patients. Overall, 71% (20/28) of the dogs had a bladder UCC, with an equal distribution of T2 and T3 tumours. Primary urethral UCC was reported in 11% (3/28) and prostatic UCC in 18% (5/28) of the population.

In the mitoxantrone-group, 71% (15/21) of the dogs had bladder UCC, 10% (2/21) urethral UCC, and 19% (4/21) prostatic UCC. Among the patients with bladder UCC, 46% (7/15) were T2 and 53% (8/15) were T3 tumour stage. Four of the 21 dogs had regional metastasis. A fifth dog had no evidence of regional metastatic disease but had multiple pulmonary lesions at initial staging. This finding was interpreted as the presence of distant metastatic disease (T2N0M1); however, sampling was declined, and a concurrent neoplasia could not be excluded.

In the vinblastine-group, 72% (5/7) of the dogs had bladder UCC, 14% (1/7) urethral UCC with prostatic involvement, and 14% (1/7) prostatic UCC. Among the patients with bladder UCC, 60% (3/5) were T2 and 40% (2/5) were T3 tumour stage. Two of the seven dogs had regional metastasis. No dogs in the vinblastine-group had detectable distant metastasis. 

Meloxicam was administered at the dose of 0.1 mg/kg PO q 24 h in all the cases. Pre-treatment with meloxicam was reported in 32% (9/28) of the studied population. In eight dogs from the mitoxantrone-group and in one dog from the vinblastine-group. Meloxicam was given for a median of 23 days (range 12–33 days) for the management of the patient’s clinical signs during the diagnostic process. The clinical response to meloxicam, as a sole therapy, was subjectively evaluated in eight of the nine patients pre-treated with meloxicam. Improvement in the lower urinary clinical signs (resolution of stranguria, urinary incontinence, and gross haematuria, as well as reduction in the urinary frequency) was reported in all eight patients prior to starting chemotherapy. There was no reported improvement in tenesmus in the patients with prostatic involvement (2/8) following pre-treatment with meloxicam.

Twenty-one dogs received mitoxantrone (5–5.5 mg/m^2^ q 21 days) and seven dogs received vinblastine (2–2.5 mg/m^2^ q 14 days). The median number of treatments was 4 (range 1–17) for the mitoxantrone-group and 8 (range 1–15) for the vinblastine-group.

**Figure 1:**
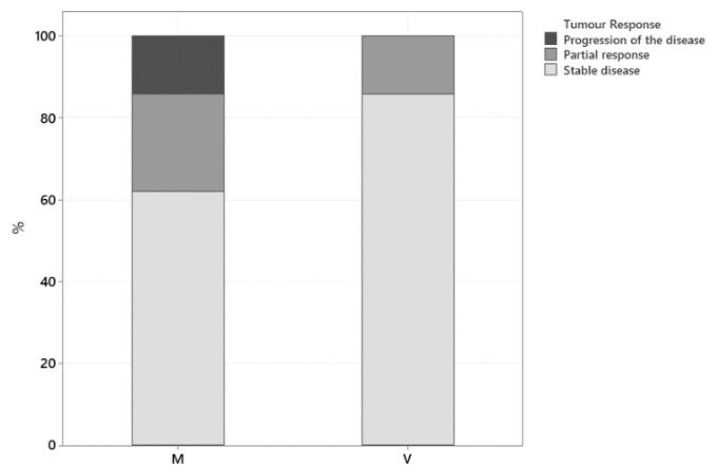
Tumour response for mitoxantrone-group (M) (*n* = 21) and vinblastine-group (V) (*n* = 7).

Restage imaging was performed two weeks following initiation of treatment in 1 dog, every 6 weeks in 16 dogs, every 8 weeks in 2 dogs, and every 12 weeks in 8 dogs. Only one dog evaluation of tumour response was based on amelioration or deterioration in clinical signs. A clinical benefit from treatment was observed in 89% of dogs (25/28), with 21% (6/28) exhibiting partial response (PR) and 68% (19/28) having stable disease (SD). Progression of the disease (PD) was observed in 11% of dogs (3/28). None of the patients included in this study achieved complete remission. Tumour response for each group is summarised in Figure 1. In the mitoxantrone-group there were 24% (5/21) PR, 62% (13/21) SD, and 14% (3/21) PD, while in the vinblastine-group there were 14% (1/7), 86% (6/7), and 0%, respectively. The two groups did not differ significantly according to a Fisher exact test (*p* = 0.529). Initial tumour response was evaluated via ultrasound in 26/28 cases and based on clinical signs in 2/28 dogs. The two dogs where tumour response was evaluated based on the improvement but no resolution of clinical signs (dysuria) were classified as stable disease until deterioration was reported after the third and sixth mitoxantrone dose, respectively.

**Figure 2:**
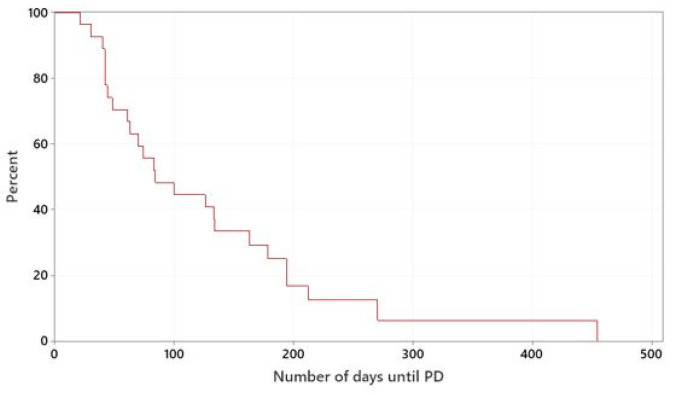
Kaplan–Meier curves plotting TTP (days) of dogs treated with meloxicam in combination with mitoxantrone or vinblastine.

The median TTP of dogs treated with meloxicam in combination with mitoxantrone or vinblastine was 84 days. The mitoxantrone-group had a median TTP of 70 days and the vinblastine-group had a median TTP of 178 days. There was no statistically significant difference between the two groups (*p* = 0.212), (Figure 2 and Figure S1).

**Figure 3:**
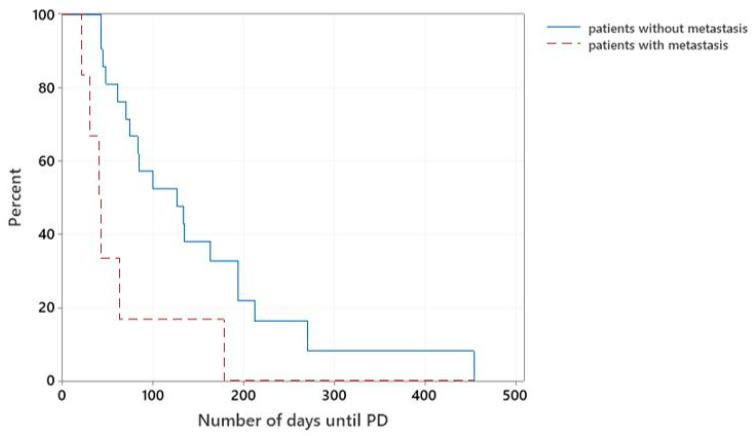
Kaplan–Meier curves plotting TTP (days) of dogs treated with meloxicam in combination with mitoxantrone or vinblastine that presented with or without metastatic disease.

Overall, those patients that were diagnosed with metastatic disease at the time of initial staging (7/28) had significantly shorter median TTP than patients without evidence of regional or distant metastasis (21/28), with a median TTP of 40 days versus 126 days, respectively (*p* = 0.007), (Figure 3).

**Figure 4:**
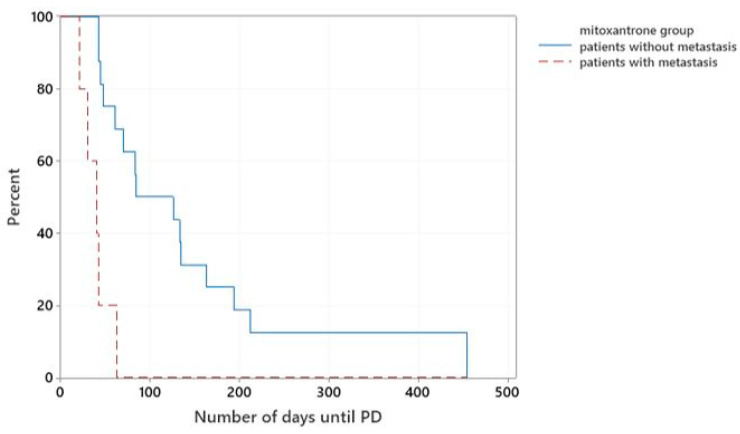
Kaplan–Meier curves plotting TTP (days) of dogs from the mitoxantrone-group that presented with or without metastatic disease.

In the mitoxantrone-group, 5/21 dogs presented with metastatic disease. The median TTP of these patients was 40 days, while patients without evidence of metastatic disease at presentation had a significantly longer median TTP of 84 days (*p* < 0.001), (Figure 4).

In the vinblastine-group, 2/7 dogs with bladder UCC presented with regional metastasis. The median TTP of these patients following vinblastine was 178 days, while those patients treated with vinblastine without evidence of metastatic disease at presentation had a median TTP of 194 days. This was not statistically significant (*p* = 0.678).

Gastrointestinal AEs occurred in 21% (6/28) of the patients during their treatment course. These are summarised in Table 2. All the 6/28 (21%) dogs were reported to have diarrhoea, 3 (11%) nausea, and 2 (7%) vomiting. Concurrent AEs were reported in 3/6 dogs with gastrointestinal AEs. The gastrointestinal AEs occurred 3–7 days following chemotherapy treatment. They were more common in the vinblastine-group (2/7; 28%) than in the mitoxantrone-group (4/21; 19%). However, this difference did not reach statistical significance (*p* = 0.622). In the mitoxantrone-group, three dogs had grade 1 diarrhoea after the first dose and one of them had concurrent grade 1 nausea; another dog had grade 3 diarrhoea, grade 1 vomiting, and grade 1 nausea after the sixth dose. In the vinblastine-group, one dog had grade 1 diarrhoea and grade 2 vomiting after the second dose; the second dog had grade 2 diarrhoea and grade 4 nausea after the first dose.

The lowest dose of chemotherapy (vinblastine 2 mg/m^2^ or mitoxantrone 5 mg/m^2^) was prescribed in 5/6 patients that presented with gastrointestinal AEs. Only one patient from the mitoxantrone-group with reported gastrointestinal AEs (grade 1 diarrhoea and nausea) received a higher dose of chemotherapy (5.5 mg/m^2^). Gastrointestinal AEs resolved after a break off meloxicam and the same chemotherapy dose was continued.

Due to gastrointestinal AEs, meloxicam was discontinued in 3/6 dogs (in two dogs of the mitoxantrone-group and one dog of the vinblastine-group), due to owners’ preference, continuing only with chemotherapy. In the remaining dogs, a break from meloxicam until resolution of the gastrointestinal AEs was prescribed and meloxicam was then reintroduced without concerns.

Haematological AEs occurred in 28.6% (6/21) of the patients treated with the combination of mitoxantrone (5 mg/m^2^) and meloxicam. Five out of six dogs developed neutropenia after the first mitoxantrone dose: one dog developed grade 1 neutropenia, three dogs developed grade 2 neutropenia, and one dog developed grade 4 neutropenia. The latter had a 20% dose reduction. However, grade 3 neutropenia was detected despite dose modification. Progression of the disease was noted at that point and treatment was then discontinued. The sixth patient developed grade 4 neutropenia after the sixth mitoxantrone dose (5 mg/m^2^) and treatment was discontinued because of the owner’s decision against further AEs. Five of the six dogs that experienced neutropoenia after mitoxantrone weighed <15 kg. Only one patient from the vinblastine-group (1/7; 14.3%) developed grade 2 neutropenia after the fourth dose of vinblastine at a dose of 3 mg/m^2^. The dose was reduced by 10% and the dog was continued on the same dose, given it was well tolerated. The weight of this patient was >15 kg. 

At the end of the study, 24 dogs had PD and 3 dogs (two of the mitoxantrone-group and one of the vinblastine-group) had SD and were still on the treatment. One dog was euthanised following the first chemotherapy dose (vinblastine) after developing acute kidney injury with no evidence of PD on abdominal ultrasound. Progression of the disease was confirmed via ultrasound in 23/24 dogs and based on clinical signs in 1/24 dogs. The patient where PD was based on deterioration of the clinical signs had a negative urine culture. In 17/25 (68%) dogs, recurrence of the lower urinary clinical signs was reported at the time of progression. In the dog diagnosed with distant metastasis (T2N0M1), increase in the frequency of coughing episodes was reported at the time of progression.

In the mitoxantrone-group, 15/21 dogs received one or more rescue therapies (carboplatin in 9 dogs, vinblastine in 7 dogs, metronomic chlorambucil in 6 dogs, and toceranib phosphate in 1 dog). Five of fifteen dogs that had a rescue treatment were lost to follow up; the rest of the patients were euthanised due to tumour progression. Two patients from the mitoxantrone-group were still alive at the end of the study: one dog was still receiving mitoxantrone as a first-line treatment and the other dog was on its fourth rescue protocol with toceranib phosphate.

In the vinblastine-group, 3/7 dogs received one rescue therapy (metronomic chlorambucil in 1 dog and carboplatin in 2 dogs). One dog that received rescue therapy was lost to follow up and the rest of the patients were euthanised due to tumour progression. One of the patients was still alive at the end of the study, receiving vinblastine as a first-line treatment. A detailed description of rescue therapies, time to tumour progression, and survival of the patients from the mitoxantrone- and vinblastine-groups are documented in Supplementary Material Tables S1 and S2, respectively.

## 4. Discussion

The purpose of this study was to assess the efficacy of meloxicam in combination with two commonly used chemotherapy agents, mitoxantrone and vinblastine, for the management of canine UCC, as well to evaluate the gastrointestinal AEs associated with these treatment combinations.

A mass at the trigone region of the bladder is the most common location for canine bladder UCC. Most of the patients (78%) present with tumour stage T2 (invading the bladder wall) at the time of diagnosis followed by tumour stage T3 presentation (invading neighbouring organs) with an incidence of 20%. Primary urethral UCC or involvement of the urethra is reported in more than 50% of the cases and prostatic UCC has an incidence of 29% [1,30]. The two latter presentations have been associated with a worse prognosis and the increased risk of development of metastatic disease [30,31]. Overall, 64% (18/28) of the dogs in this study presented with a bladder (stage T3) (10/28), urethral (3/28), or prostatic (5/28) UCC. The incidence of regional metastasis at the time of diagnosis was 21%, which is similar to the incidence of metastasis previously described for this tumour type (16%) [2,30]. The incidence of distant metastasis at the time of diagnosis was 4%, which was reported to be between 14 and 20% in other studies [1,2].

In one study by Henry et al., evaluating mitoxantrone and piroxicam for the management of canine UCC, the tumour stage of the studied population was predominantly T2, with only 14% of patients presenting with T3 stage. Primary urethral or prostatic UCCs were not reported, and metastatic disease was present in 11% of the patients at the time of diagnosis [19]. In another study by Allstadt et al., assessing the same treatment combination, the tumour stage of the enrolled population was similar to the one reported in this study. More advanced disease (tumour stage T3, urethral or prostatic UCC) was reported in 54% of the patients. However, only 8% of the patients had metastatic disease at the time of diagnosis [18]. In the current study, in the mitoxantrone-group, 14/21 (67%) of the patients had a more advanced tumour stage, with 8/21 (38%) dogs presenting with T3 UCC and 6/21 (28.5%) dogs presenting with urethral or prostatic UCC (2 and 4 dogs, respectively). The incidence of regional metastasis at the time of diagnosis was 19% (4/21) and the incidence of distant metastasis, which was based on imaging assessment without cytological or histopathological confirmation, was 6% (1/18).

The presence of metastasis at the time of diagnosis has been described as a negative prognostic factor [30,32]. In this study, those patients that presented with metastatic disease at the time of diagnosis also had a shorter TTP than those patients that presented without evidence of metastatic disease. The time to progression (TTP) associated with the treatment combination of mitoxantrone and meloxicam was assessed for the first time in this study, and it was shorter than the TTP reported in the study by Henry et al., which assessed the combination of mitoxantrone and piroxicam (70 vs. 194 days) [19]. This could have been influenced by the higher number of patients presenting with tumour stage T3, metastatic disease at the time of diagnosis, and involvement of the urethra and prostate, compared to the previous study population [19]. TTP was similar to that reported in the second published study by Allstadt et al., which assessed the combination of mitoxantrone and piroxicam (70 days vs. 106 days). In this latter study, the studied population was similar in regard to the extent of the disease [18]. 

The clinical benefit (complete response, partial response, and stable disease) obtained with the combination of mitoxantrone and meloxicam in our study was 86%, which is similar to the clinical benefit reported with the combination of mitoxantrone and piroxicam in previous studies (77% [18], and 81% [19]). 

In the vinblastine-group, 57% of the patients had a more advanced tumour stage with 2/7 (28.5%) dogs presenting with T3 UCC and 2/7 (28.5%) dogs presenting with urethral or prostatic UCC (1 of each). This differs from the previous study assessing vinblastine in combination with piroxicam, where 25% of the population had T3 UCC with no primary urethral or prostatic presentation [16]. The incidence of regional metastatic disease was also higher in the current study (29%) in comparison to the previous study by Knapp et al., which assessed vinblastine alongside piroxicam, where none of the patients presented with regional metastasis. In that study, only one patient was diagnosed with distant metastatic disease (4%) [16].

The CB and TTP obtained from the combination treatment of vinblastine and meloxicam were assessed for the first time, and the results are similar to those reported with the combination treatment of vinblastine and piroxicam (100% and 178 days vs. 92% and 199 days, respectively) [16]. 

Vinblastine as a single agent for the management of canine UCC has been assessed in two different studies with similar CB but shorter TTP than those reported in combination with piroxicam. The CB reported was 86–93% and TTP was 122–143 days [16,32]. The population of dogs in these studies differed from the vinblastine-group in the current study in regard to tumour location, tumour, and clinical stage. Therefore, further studies with larger and more homogenous populations, comparing the tumour response of patients treated with the combination of vinblastine and meloxicam, meloxicam as a sole therapy, and vinblastine as a first-line treatment would be needed to determine the benefit of this treatment combination. The incidence of gastrointestinal AEs in the mitoxantrone-group (19%) was similar to that previously reported with mitoxantrone and piroxicam treatment combination (18%) [19]. However, the severity of the gastrointestinal AEs was lower when compared to that in previous studies, as supportive medication or hospitalisation was not required for its resolution [18,19]. The incidence of gastrointestinal AEs for the vinblastine and meloxicam treatment combination was roughly similar (28.5%) to that reported with vinblastine in combination with piroxicam (21%), with similar severity of gastrointestinal AEs, i.e., mainly VCOG grade 1–2 [16]. In the same study, the gastrointestinal toxicity of vinblastine as a single agent was reported to be lower than that in combination with piroxicam (18.5%), being mainly VCOG grade 1 [16].

The therapeutic effects of NSAIDs are dependent on COX-2 inhibition, whereas AEs result from COX-1 inhibition by blocking PGs synthesis. Therefore, the long-term use of conventional NSAIDs (which inhibit both COX-1 and COX-2), such as piroxicam, is known to be associated with gastrointestinal and renal AEs [3,4,5,6]. Selective COX-2 inhibition does not influence the prostaglandin synthesis of the gastric mucosa, which is mainly produced by the COX-1 enzyme [33]. This could explain why conventional NSAIDs (non-selective COX-2) are related to more gastrointestinal side effects in comparison to selective COX-2, such as meloxicam.

Based on the owners’ observations, eight of the patients pre-treated with meloxicam improved their lower urinary clinical signs prior to starting chemotherapy. This is likely to be due to the anti-inflammatory and/or antitumoral activity of meloxicam previously reported with this tumour type.

In our study, 71.4% (5/7) of the dogs that developed neutropenia had a bodyweight of <15 kg; four of the patients were treated with mitoxantrone. Previous studies have reported that smaller dogs exhibited higher levels of chemotherapy-induced neutropenia than larger dogs [34], which is in line with our findings. The epithelium of canine UCC has shown an overexpression of COX-2 (90%) and LOX-5 (95%) enzymes [8,35]. COX-1 has also been detected in canine UCC, but in lower numbers [8]. Therefore, the use of meloxicam, a readily available selective COX-2, should also be effective as a sole therapy, as seen with other selective COX-2 inhibitors, including deracoxib and firocoxib; however, this requires further evaluation.

The main limitations of this study are its retrospective nature, the heterogenous and small sample size, the lack of a control group with patients treated only with meloxicam, and the lack of standardised staging/restaging modality and its frequency. The two latter factors could have biased the TTP obtained in our study. The lack of a control group with patients treated only with meloxicam made it difficult to evaluate the antineoplastic effect of this selective COX-2 inhibitor. However, the subjective improvement in clinical signs described in dogs treated with meloxicam alone, prior to chemotherapy, would suggest that this NSAID plays a positive role in the management of UCC. The participation of different institutions influences the decisions taken regarding management and treatments, and determining tumour response. In the two dogs where tumour response was based on the evaluation of clinical signs, this may have resulted in an inaccurate evaluation. The presence of pre-treated animals with meloxicam could also interfere with tumour response and time to progression. Therefore, this risk was minimised by excluding those patients pre-treated with other NSAIDs or meloxicam for more than 6 weeks prior to chemotherapy.

## 5. Conclusions

The results of this study suggest that meloxicam in combination with mitoxantrone or vinblastine are well-tolerated first-line treatment options for non-resectable canine UCC. They offer similar clinical benefits as in combination with piroxicam. This suggests that meloxicam is a potential alternative NSAID for the treatment of canine UCC. However, further studies with a larger sample size and a control group would be needed to confirm these findings and to assess the incidence of non-gastrointestinal AEs.

## Figures and Tables

**Table 1:** Characteristics of 28 dogs with UCC and comparison of those between mitoxantrone and vinblastine-group.

Characteristics	Whole Population (*n* = 28)	Mitoxantrone-Group (*n* = 21)	Vinblastine-Group (*n* = 7)	*p*-Value
Mean age (years)	10.5 (5–15)	10.7 (5–15)	10.2 (9–14)	0.750
Sex				1.000
FN	11 (39%)	8 (38%)	3 (43%)	
MN	16 (57%)	12 (57%)	4 (57%)	
ME	1 (4%)	1 (5%)	0 (0%)	
Mean weight (Kg)	12.5 (3.7–37)	12 (3.7–37)	23.4 (5–37)	0.937
Tumour location				
Bladder	20 (71%)	15 (71%)	5 (72%)	
Urethra	3 (11%)	2 (10%)	1 (14%)	
Prostate	5 (18%)	4 (19%)	1 (14%)	
Stage				
Metastasis				
Regional	6 (22%)	4 (19%)	2 (29%)	
Distant	1/23 (4%)	1/18 (6%)	0/5 (0%)	

Abbreviations: FN: female neutered, MN: male neutered, ME: male entire.

**Table 2:** Gastrointestinal AEs associated with mitoxantrone-group (M) and vinblastine-group (V).

	M (*n* = 4)				V (*n* = 2)	
Dogs Gastrointestinal AEs	1	2	3	4	1	2
Grade 1 diarrhoea	x	x	x		x	
Grade 2 diarrhoea						x
Grade 3 diarrhoea				x		
Grade 1 nausea			x	x		
Grade 4 nausea						x
Grade 1 vomiting				x		
Grade 2 vomiting					x	

Abbreviations: M: mitoxantrone-group, V: vinblastine-group, AE: adverse effects.

## Data Availability

Raw data can be made available, upon reasonable request.

## Supplementary Materials

The following supporting information can be downloaded at: https://www.mdpi.com/article/10.3390/vetsci10080529/s1, Figure S1: Kaplan–Meier curves plotting TTP (days) of dogs from the mitoxantrone-group (*n* = 21) and dogs from the vinblastine-group (*n* = 7); Table S1: rescue therapies, time to tumour progression, and survival of the patients from the mitoxantrone-group; Table S2: rescue therapies, time to tumour progression, and survival of the patients from the vinblastine-group.
