# Supplementary material for: Meloxicam in Combination with Mitoxantrone or Vinblastine as First-Line Treatment for Non-Resectable Urothelial Cell Carcinoma in Dogs
Source: Vet Sci. 2023 Aug 21;10(8):529. doi: 10.3390/vetsci10080529 (PMC10458788; doi:10.3390/vetsci10080529)
Supplement: Supplementary file 1 [file vetsci-10-00529-s001.zip › vetsci-2469170-supplementary.pdf]

Table S1: Rescue therapies, time to tumour progression and survivals of the patients from the mitoxantrone-group.

Mitoxantrone-group (n=21)	1st rescue protocol	2nd rescue protocol	3rd rescue protocol	4th rescue protocol	Time to tumour progression (TTP)	Survival time
1	x	x	x	x	163	Euthanasia (192 days)
2	vinblastine	carboplatin	x	x	44	LTFU
3	x	x	x	x	42	LTFU
4	carboplatin	vinblastine	x	x	126	LTFU
5	vinblastine	metronomic chlorambucil	x	x	70	Euthanasia (357 days)
6	carboplatin	metronomic chlorambucil	x	x	133	LTFU
7	carboplatin	x	x	x	40	LTFU
8	vinblastine	carboplatin	x	x	84	Euthanasia (151 days)
9	carboplatin	vinblastine	metronomic chlorambucil	X	83	Euthanasia (1007 days)
10	metronomic chlorambucil	x	x	x	30	Euthanasia (157 days)
11	vinblastine	x	x	x	21	Euthanasia (176 days)
12	x	x	x	x	454	Euthanasia (507 days)
13	vinblastine	carboplatin	metronomic chlorambucil	toceranib phosphate	61	Still alive (343 days)
14	x	x	x	x	N/A	Still alive (242 days)
15	x	x	x	x	42	Euthanasia (93 days)
16	x	x	x	x	48	Euthanasia (50 days)
17	carboplatin	x	x	x	194	Euthanasia (215 days)
18	carboplatin	vinblastine	x	x	63	Euthanasia (201 days)
19	carboplatin	x	x	x	42	Euthanasia (71 days)
20	x	x	x	x	134 ** Based on clinical signs	Euthanasia (135 days)
21	metronomic chlorambucil	x	x	x	212	LTFU

Abbreviations: N/A: non-applicable, LTFU: loss to follow up

Table S2: Rescue therapies, time to tumour progression and survivals of the patients from the vinblastine-group.

Vinblastine-group (n=7)	1st rescue protocol	2nd rescue protocol	3rd rescue protocol	4th rescue protocol	Time to tumour progression (TTP)	Survival time
1	metronomic chlorambucil	x	x	x	178	Euthanasia (301 days)
2	x	x	x	x	270	LTFU
3	carboplatin	x	x	x	100	Euthanasia (106 days)
4	x	x	x	x	74	Euthanasia (163 days)
5	x	x	x	x	N/A	Euthanasia (14 days) - AKI
6	carboplatin	x	x	x	194	LTFU
7	x	x	x	x	N/A	Still alive (158 days)

Abbreviations: N/A: non-applicable, LTFU: loss to follow up, AKI: acute kidney injury
